# Massive subcutaneous emphysema after traumatic pneumothorax

**DOI:** 10.11604/pamj.2016.23.56.8947

**Published:** 2016-02-29

**Authors:** Adriá Rosat, Pilar Gómez

**Affiliations:** 1 Department of General Surgery, Hospital Universitario Nuestra Señora de Candelaria, Ctra, Del Rosario 145, 38010 Sta, Cruz de Tenerife, Spain; 2 Department of Internal Medicine, Hospital Universitario Nuestra Señora de Candelaria, Ctra, Del Rosario 145, 38010 Sta, Cruz de Tenerife, Spain

**Keywords:** Massive subcutaneous, emphysema, pneumothorax

## Image in medicine

A 52-year-old man was admitted to our hospital emergency room for dyspnea and extensive chest swelling. He had a right chest trauma after falling over the bathtub. He was hemodynamically stable and on physical examination he had right chest hypoventilation with hypoxemia. The swelling quickly progressed to the cervical region and the skin crackled. Chest X-ray revealed a right pneumothorax with massive subcutaneous emphysema. He was treated by chest drainage and both the emphysema and the pneumothorax completely resolved several days later. Subcutaneous emphysema is usually a benign, self-limiting condition requiring only conservative management. The main risk of subcutaneous emphysema is the massive accumulation of air in the deeper tissue planes that can compromise the life of the patient. This accumulation can substantially compress the trachea and the great vessels, which can severely compromise the airway, venous return, and blood flow to the head and neck. Many techniques have been shown to provide effective treatment. Placing a chest tube and increasing the suction is the most effective and safe option in patients developing subcutaneous emphysema following pneumothorax. Insertion of a drain into the subcutaneous tissue provides rapid relief, aided or not by regular compressive massage. Infraclavicular incisions are also effective, but are noted to be more invasive and carry the potential for cosmetic defect. The optimal approach to patients who present with subcutaneous emphysema is not always apparent. A watchful-waiting attitude is recommended in limited subcutaneous emphysema. Indications must be carefully considered on an individual patient basis.

**Figure 1:**
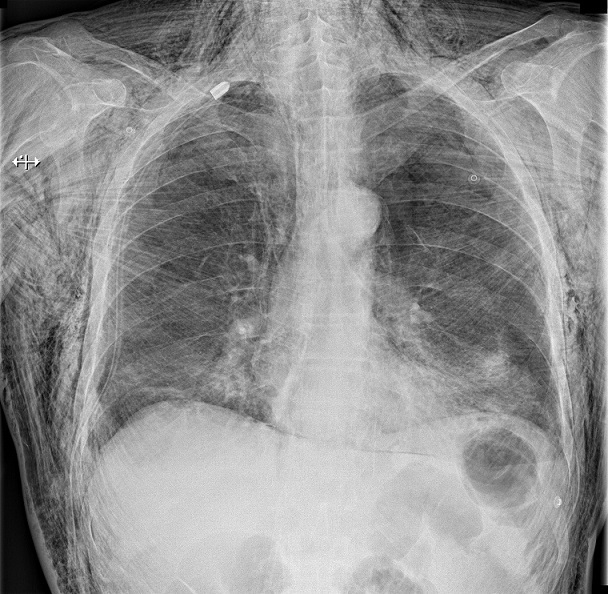
Chest x-ray showing a massive subcutaneous emphysema. Right lung reexpansion after chest tube placement

